# Prolonged Cholestasis Following Acute Hepatitis A Infection: Case Report and a Review of Literature

**DOI:** 10.7759/cureus.38511

**Published:** 2023-05-03

**Authors:** Mohamad B Alebaji, Alaa S Mehair, Ola I Shahrour, Feryal A Elkhatib, Eiman H Alkaabi, Najla S Alkuwaiti

**Affiliations:** 1 Pediatric Medicine, Tawam Hospital, Abu Dhabi, ARE

**Keywords:** pediatric weight loss, jaundice cholestatic, abdominal pain without diarrhea, recurrent cholestasis, viral hepatitis a

## Abstract

Prolonged cholestasis is a rare complication of hepatitis A infection, characterized by a protracted, indolent course with persistent symptoms of pruritus and malabsorption lasting for several months. A 14-year-old girl, previously treated for acute hepatitis A, presented with yellowing of the eyes. An evaluation of her liver function revealed conjugated hyperbilirubinemia, and a liver biopsy confirmed hepatitis with cholestasis. Fortunately, she responded well to conservative treatment and recovered successfully.

## Introduction

Hepatitis A is the most prevalent form of viral hepatitis in underdeveloped nations, and it is a mostly asymptomatic and self-limiting illness [1]. The virus is present in the liver, bile, feces, and blood during the late incubation (median 28 days) period and acute pre-icteric phase of the disease. However, viral shedding in feces, viremia, and infectivity decrease quickly once jaundice becomes evident. While rare, hepatitis A can lead to complications, with the most severe consequence being fulminant hepatic failure, which can occur if co-infection with the hepatitis E virus is also present [2]. Although rare, a fulminant form of the disease can manifest in adults [3], along with other unusual clinical types such as relapsing hepatitis, protracted hepatitis, and cholestatic hepatitis [4].

Cholestatic hepatitis, marked by continuously elevated bilirubin levels of more than 5 mg/dl for more than four weeks, is a rare complication of hepatitis A, accounting for between 0.8% and 5.2% of hepatitis A patients [5-7]. It is characterized by symptoms such as pruritus, lethargy, diarrhea, and weight loss. As the reported instances of hepatitis A continue to rise, so does the number of individuals with cholestatic hepatitis caused by the virus. The medical literature describes different therapeutic strategies for treating cholestatic hepatitis [8]. In this article, we present a case report of cholestasis caused by hepatitis A infection, which was treated conservatively.

## Case presentation

We are reporting on the case of a 14-year-old Pakistani adolescent girl who presented to our hospital with a 10-day history of high-grade fever (max 103 F) accompanied by chills, vomiting, and epigastric pain. She also had yellowish skin discoloration and had been passing brown urine for the past five days prior to admission. The patient had arrived from Pakistan 12 days before the presentation, and her older sister had been admitted to the hospital with the same symptoms a month prior and was found to be hepatitis A positive. The patient had no significant past medical history, and her family members were healthy. There was no history of blood transfusions or prescribed medication.

On examination, the patient was icteric and febrile but hemodynamically stable. Her abdomen was tender at the epigastric and right upper quadrant regions with no guarding or rigidity. No hepatosplenomegaly or lymphadenopathy was noted. Laboratory investigations showed deranged liver function tests (Table 1).

**Table 1:** Liver function test during the course of admissions ALP - alkaline phosphatase, GGT - gamma-glutamyl transferase, AST - aspartate aminotransferase, ALT - alanine transaminase

Liver function test	First admission		Readmission							Follow-up
	On admission	Discharge	Day 1	Day 2	Day 4	Day 6	Day 15	Day 20	Day 22	Day 36
Total protein (66 g/L - 87 g/L)	76	68	74	73	72	68	62	-	63	68
Albumin (35 g/L - 52 g/L)	31	25	26	25	25	25	22	-	21	24
Bilirubin total (<17 micromol/L)	200.7	146	512	520.3	510.4	547.6	439	463	373	136.2
Bilirubin direct (<5.0 Micromol/L)	177.6	134	447.4	459.7	461.1	461.5	391	376.3	347	131.1
ALP (35 IU/L - 104 IU/L)	304	254	188	191	187	187	194	212	192	158
GGT (4 IU/L - 24 IU/L)	177	172	-	-	14	14	20	24	-	
AST (<32 IU/L)	556	302	71	66	50	49	68	81	52	61
ALT (<32 IU/L)	1125	1665	60	54	43	33	35	55	37	31

A hepatitis panel showed positive hepatitis A IgM antibodies. The complete blood count and coagulation profile were normal. She was treated conservatively as a case of acute hepatitis A and showed clinical improvement with a drop in liver enzymes. The patient was discharged home with supportive instructions.

However, after seven days, she returned to the emergency department with progressively worsening yellowish discoloration involving her skin, eyes, nails, and hands, along with a burning sensation in her feet. She also reported a mild headache associated with dizziness but denied confusion, abnormal movement, or any decrease in activity or oral intake. Vital signs were stable, and she was afebrile. Repeated labs revealed further elevation of liver enzymes and worsening of direct hyperbilirubinemia (Table 1). A repeated hepatitis profile showed positive hepatitis A IgM. Thus, she was readmitted as a case of prolonged direct hyperbilirubinemia.

Due to the prolonged course of elevated liver enzymes, the suspicion of additional underlying chronic disease was entertained. The gastroenterology team was informed, and further investigations were done. Epstein-Barr virus (EBV) IgM and IgG (both positive) and *Mycoplasma pneumoniae* IgM (positive) were found. In view of the positive result for *Mycoplasma*, autoimmune hepatitis was considered but eventually eliminated as the serum levels of anti-LKM antibody (anti-liver-kidney microsomal antibody, or LKM antibody), smooth muscles antibodies (SMA), and antinuclear antibody (ANA) were sent and came back negative. Abdominal ultrasound was normal, with no gallbladder stones noted. There was no evidence of stones or changes in CBD or intrahepatic ducts on MRI liver or MRCP (magnetic resonance cholangiopancreatography).

Due to the persistent cholestasis, the case was investigated for Wilson's disease. Accordingly, urine containing copper was collected, and an eye examination was done by an ophthalmologist. No Kayser Fleischer ring was seen. Eventually, a liver biopsy was done, and the report noted marked cholestasis with a mild portal and lobular inflammation and focal bridging fibrosis. No evidence of Wilson's disease or other chronic disorders was present. A series of closely monitored liver enzymes revealed initially stable levels, but around 36 weeks after presentation, there was a significant decrease in the bilirubin level (Table 1).

## Discussion

Globally, over two million cases of hepatitis A infection are diagnosed each year. Although seroprevalence and infection rates have fallen in the United Arab Emirates (UAE), the majority of infections originate in other endemic nations [9].

One of the unusual signs of hepatitis A infection is prolonged cholestasis. Proper diagnosis and treatment of this manifestation are necessary because it can increase morbidity and health costs [10]. Prolonged cholestasis is defined as increased total bilirubin >5 mg/dL for more than four weeks following diagnosis [11]. Patients with acute hepatitis A and cholestatic hepatitis experience intrahepatic cholestasis, persistent jaundice, appetite loss, itching, dark-colored urine, and gray-colored feces. Additionally, total bilirubin, direct bilirubin, alkaline phosphatase (ALP), and gamma-glutamyl transferase (GGT) levels increase in cholestatic hepatitis patients. Our case had the same symptoms during presentation.

In hepatitis A, intrahepatic cholestasis occurs between 0.4% and 0.8% of the time. The incidence of protracted cholestasis owing to hepatitis A infection with a bilirubin level of 8-38 mg/dl was found to be 7.0% in prior research and 4.7% in a subsequent investigation conducted in Korea [12].

Cholestasis is caused by a variety of mechanisms, such as diminished bilirubin absorption, conjugation malfunction, bilirubin excretion dysfunction, and biliary blockage. Cholestasis occurs in hepatitis A due to an inflammatory process. The liver secretes endotoxin and pro-inflammatory cytokines, leading to a systemic reaction [13].

This condition does not impact the liver's synthetic functions and often resolves without leaving any lasting effects. However, if the patient continues to have severe symptoms of pruritus and fat malabsorption despite ursodeoxycholic acid treatment, a course of steroids can be administered [14].

There are many case reports which showed hepatitis A virus-associated prolonged cholestatic jaundice successfully treated with oral steroids, but its role is still controversial. One report described a 12-year-old boy with prolonged cholestatic hepatitis who did not respond to cholestyramine and rifampicin but improved with oral prednisolone, which was initiated around the eighth week of presentation due to recalcitrant pruritus [15]. In our patient, oral steroids were not started as the pruritus settled with supportive treatment only. We treated our case with supportive medication, and the patient recovered successfully.

## Conclusions

This case highlights the need for a comprehensive evaluation of patients with persistent jaundice after hepatitis A infection, particularly in areas with high endemicity. A delay in diagnosis and treatment may result in significant morbidity and mortality. Therefore, clinicians should be aware of the potential for prolonged cholestasis in these patients and consider a thorough evaluation, including liver function tests and imaging studies, to aid in early diagnosis and management. Timely and appropriate treatment can improve outcomes and prevent complications associated with prolonged cholestasis.
